# Establishing the first institutional animal care and use committee in Egypt

**DOI:** 10.1186/s13010-016-0035-3

**Published:** 2016-04-09

**Authors:** Sohair R. Fahmy, Khadiga Gaafar

**Affiliations:** Zoology Department of Zoology, Faculty of Science, Cairo University, Giza, 12613 Egypt

## Abstract

**Background:**

Although animal research ethics committees (AREC) are well established in Western countries, this field is weakly developed and its concept is poorly understood in the Middle East and North Africa region.

**Objective:**

Our main objective was to introduce the concept and requirements of ethical approaches in dealing with experimental animal in research and teaching in Egypt.

**Methods:**

Due to its very recent inception, Cairo University, Faculty of Science IACUC decided to operate in accordance with Guide for the Care and Use of Laboratory Animals 8th Edition 2011 (the Guide) since Egypt has not yet compiled its own guide.

**Results:**

Fifty protocols were reviewed in 2013–2014. Only ten protocols were reviewed in 2013, but in 2014, forty protocols were reviewed. In 2013 all protocols were approved and in 2014, number of approvals were 35, the number of deferrals were 4, and one refused protocol. Master’s theses (MSc) research protocols constituted the majority of the total reviewed protocols. This is attributed to the decision of the Board of the Faculty of Science, Cairo University in September, 2013 that the approval of the IACUC is mandatory before conducting any research involving animals or theses registration.

**Conclusion:**

The first IACUC was established in the Cairo University, Faculty of Science, since 2012. The challenges encountered by the committee were diverse, such as the absence of laws that control the use of animal models in scientific research, lack of guidelines (protocols for experimental animals in research) and, mandatory ethical approval for any experimental animal research.

## Background

Although in Egypt committees for the ethical considerations on human research issues have been established for many years, committees for animals used in research ethical issues were not that fortunate. The human medical research ethics committees are referred to as either Institutional Review Board (IRB) or Research Ethics Committee (REC). Some of the RECs may approve animal research protocols when needed such as the Medical Research Ethics Committee (MREC) of the National Research Center (NRC) [1]. Cairo University has been the incubator and disseminator of knowledge and culture in Egypt and the neighboring countries for the last century. Since it was founded in 1925, the Faculty of Science has been the motivator of scientific achievements and vision, where many generations of outstanding scientists have graduated. In the last decade, it has been paying great attention to high quality international animal research standards, thereby necessitating the standardization of the care and use of the experimental animals to achieve global recognition. The assurance that the caliber of animal research and animal welfare are consistent and that such animal use is done in a humane and conscientious manner is of concern to the scientific community, the general public, and other stakeholders.

The care and use of animals play a critical role to the scientists engaged in biomedical research. Standardizing animal care and use practices is vital due to the international scientific need for reproducibility and statistical validity of results essential to quality research [2].

## Laws and regulations in Egypt

Egypt was the first country in the Middle East region to enforce regulations concerning animal welfare [3]. The official decrees of the Ministry of Agriculture in Egypt relevant to animal welfare are No. 27 (1967) that enforces the humane treatment of animals in general, while No. 45 (1967) concerns animal slaughter [4]. In the 2014 Egyptian constitution, article 45 guarantees the humane treatment of animals and protects those under threat of extinction or danger (sis.gov.eg) [5]. Until now there are no decrees in Egypt that govern the use and care of experimental animals in research and teaching.

Egypt is an active participant in the World Organization for Animal Health (OIE) and was one of the first signatories of the mandate governing animal welfare. Egypt organized the second OIE Global Conference (October 2008) on Animal Welfare “Putting the OIE Standards to Work” for the implementation of animal welfare standards worldwide and assisting developing countries in this field. The first conference was held in Paris (2004), the OIEʼs mother home country, which paved the way for the unanimous adoption of global animal welfare standards by OIE Members. Since the early 2000s the OIE decided to develop the first international standards on the care and use of animal in research and education. At the OIE 78th General Assembly (2010), Terrestrial Code Chapter 7.8, which deals with the experimental animal care and use, states the obligatory establishment of institutional animal care and use committees (IACUC) in the 177 OIE Member Countries.

The difference between animal welfare and animal rights is not well discerned in Egypt, thereby the use of the word “welfare” is misleading and usually “rights” is meant. In Egypt, animal welfare is the responsibility of the Official Veterinary Services. Their activities are limited to health care, treatment, vaccination, feeding and housing, etc. There are 13 animal “welfare” non-governmental organizations (NGOs) presently registered. Their main mission is to help abandoned and stray animals. The outcomes of their efforts are not well known due to lack of coordination.

Since Islam is the predominant religion in Egypt, animal welfare regulations are in accordance with Islamic religious teachings. In its holy book, the Quran, several verses are concerned with the importance of the humane treatment of animals. Moreover, there are several sayings of the Prophet Mohamed emphasizing the same principal. El Azhar El Shareef, the largest organization in the Middle East region dealing with Muslim affairs, established an animal welfare center to expand animal welfare awareness and to support the crucial and positive steps taken by governments or animal welfare societies towards developing guidelines on animal welfare [3].

## Guidelines used

In 1956, the International Council for Laboratory Animals Science (ICLAS) defined and established international guiding principles for using animals in biomedical research [6]. It stipulated that each institute involved in animal research should have an ethics committee for monitoring research activities on the animals. ICLAS also formed the National Accreditation Board of Testing and Calibration Laboratories to accredit Laboratories involved in animal research.

Several international guidelines for use and care of animals in scientific procedures are well established such as the Canadian Council on Animal Care (CCAC) Volume 1 [7]; the National Research Council’s Guide for the Care and Use of Laboratory Animals [8]; New Zealand’s Good Practice Guide for the Use of Animals in Research, Testing and Teaching [9]; the U.S. Public Health Service Policy on Humane Care and Use of Research Animals [10], the Australian Code of Practice for the Care and Use of Animals for Scientific Procedures [11]; and National Advisory Committee for Laboratory Animal Research (NACLAR) Guidelines on the Care and Use of Animals for Scientific Purposes [12]. Due to its very recent inception, Cairo University, Faculty of Science IACUC decided to operate in accordance with Guide for the Care and Use of Laboratory Animals 8th Edition 2011 (the Guide) since Egypt has not yet compiled its own guide.

## Training and workshops

Following establishment of Cairo University, Faculty of Science IACUC, two members of the committee attended the IACUC conference held in Baltimore, 18–19 March, 2013. They presented a poster entitled “Towards Establishing an Ethical Committee for Animal Research: Challenges and Opportunities” [13]. Other committee members got their training through workshops. Research ethics workshops were held in Cairo University to create awareness of international guidelines for animal care and use. Five workshops were conducted: (1) “Ethics in Science” in November 2012, (2) “Institutional Review Board (IRB) in Biomedical Research” (3) “Importance of Institutional Animal Care and Use Committee (IACUC)in Science” in December 2012, (4) “Guidelines of animal care and use committee” in March 2013 and (5) “Establishment of the animal care and use program” in April 2013. Other workshops were held in Faculty of Science of different universities in Egypt; Tanta University (2012), El Mansoura University (2013) and Helwan University (2014).

## Standard Operating Procedures (SOPs)

The objective of Standard Operating Procedures (SOPs) is to ensure quality and consistency in review of research proposals, to prevent infliction of unnecessary pain and sufferings before, during and after experiments on animals and to follow the Guide for the Care and Use of Laboratory Animals 8th Edition 2011 (the Guide).

## Constitution of the IACUC

The IACUC is constituted to ensure a) competent review of the ethical aspects of the research and b) independence from influences that could affect the performance of unbiased reviews.

Prior to 2012, the care for animals used in research in Cairo University, Faculty of Science was directly the responsibility of the researchers and thereby, the quality of animal care and animal welfare varied tremendously. Even within the same scientific team, research laboratories had inconsistent animal care policies and standards. In 2012, Cairo University, Faculty of Science, established its own institutional animal care and use committee (IACUC) which is responsible for (1) reviewing research protocols that require the use of animals to make sure the methods of animal care and use are compliant to the international guidelines and the Guide; (2) submitting its well considered decision and modifications if any (3) issuing an approval for commencement of research (4) inspecting animal facilities and (5) evaluating animal care programs twice a year.

The first Chairperson, who initiated the establishment of the IACUC, was appointed directly by the Dean of the Faculty of Science, Cairo University. An initial group of members were selected directly by the Chairperson. The Chairperson then seeked the approval of the Dean of the Faculty of Science, Cairo University and its Board for the whole composition of the committee. The succeeding Chairperson will be chosen by the Dean and appointed after approval of the Faculty Board.

There are thirteen members in the committee representing a multidisciplinary and multisectorial composition. Due to lack of specialties in the animal care field, the IACUC includes a number of practicing scientists experienced in research ethics committees (RECs) to enhance the reviewing process. The IACUC members include, affiliated and non-affiliated practicing scientists experienced in research involving animals, a Muslim sheikh, a veterinarian and a layperson. An IACUC member does not participate in the review or approval of a proposal in which that member has a conflicting interest.

In the first year of its establishment (2012), the IACUC had no room, secretary, computers, website or any basic facilities. The IACUC members developed a number of different application forms for reviewing a proposed research project’s observance to the international guidelines governing the use of animals in research. In 2013, an office was allocated to the committee’s activities with one computer and secretary. Moreover a website (ACUC.sci.cu.edu.eg) was developed. Regarding the budget, there is no yearly budget for the committee and there are no fees charged for protocol review.

## Meeting frequency

The IACUC meets once a month on a regularly scheduled day. The quorum requirement is half of the members with a minimum of five. In the beginning, the IACUC meeting average duration was one hour as only one or two protocols were submitted to the committee for reviewing. This increased gradually to 3 and 5 h as the researchers in the Faculty were forced to seek the IACUC’s approval for their articles to be accepted by international peer- reviewed journals.

## Voting and decision-making

All members attending the meeting while a protocol was discussed participated in the voting unless a member had a conflict of interest. One of four different decisions can be taken by the IACUC committee after reviewing a protocol: (1) Approval of research; (2) Approval with minor changes; (3) Deferral and (4) Disapproved.

## Review of applications of new studies

The IACUC members decided to review with a full committee review method to ensure the cooperation and provide all committee members with a hand’s on training. IACUC chairperson assigns one or two members to conduct a pre-review of animal use protocol applications, search for research duplication and to present the protocol for discussion at the meeting. They also help the researchers in filling the application form and guide them in fulfilling the requirements for committee approval of their protocols. All members receive protocols for review at least 1 week prior to the review meeting. They are required to review all submitted materials and be prepared to discuss all protocols at the convened meeting. All protocols are reviewed during the meeting. The secretary attends the meeting and records all notes and comments of the members, also distributes then collects the assigned voting form for each protocol. Written notification of the committee decision is sent to the researcher.

The committee undertook many training workshops to elucidate and demonstrate to the researchers how to fill their proposals (application form), and fulfill the ethical requirements to attain the approval of the committee.

The most important criteria in reviewing process include scientific design of the study, assessment of predictable risks/harms to the animals, protocol and perform of the study, plans for data analysis and reporting as well as facilities and infrastructure in the animal house. A couple of committee members established Standard Operating Procedures (SOPs) to ensure quality and consistency in reviewing of research proposals. Copy of SOPs in Arabic language was also written and distributed to researchers as a guide for the committee reviewing system. The Committee reports its findings and plans for correction of deficiencies to the Dean of the Faculty (Institutional Official (IO)).

## IACUC feedback

**Fig. 1:**
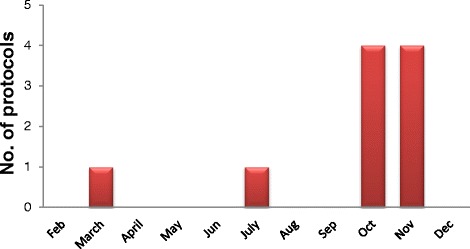
Number of revised protocols in 2013

**Fig. 2:**
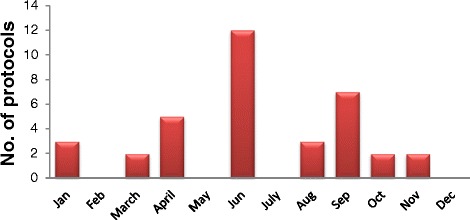
Number of revised protocols in 2014

**Fig. 3:**
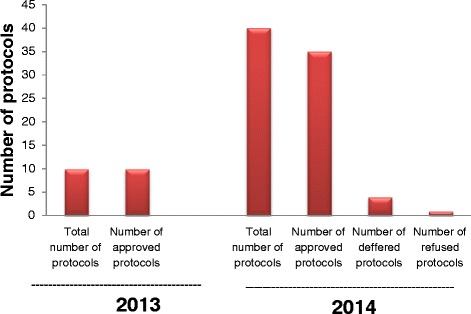
Number of approved, deffered and refused protocols in 2013–2014

Fifty protocols were reviewed in 2013–2014. Only ten protocols were reviewed in 2013 (Fig. 1), but in 2014, forty protocols were reviewed (Fig. 2). In 2013 all protocols were approved (Fig. 3) and in 2014, number of approvals were35, the number of deferrals were 4, and one refused protocol (Fig. 3).

**Fig. 4:**
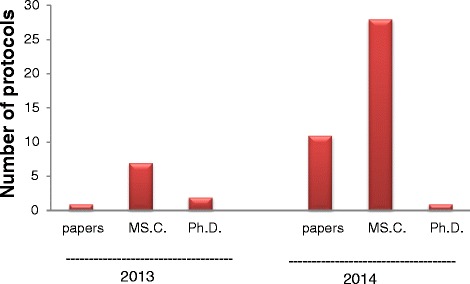
Types of revised protocols

As shown in Fig. 4, MSc research protocols constituted the majority of the total reviewed protocols in 2013 and 2014. This is attributed to the decision of the Board of the Faculty of Science, Cairo University in September, 2013 that the approval of the IACUC is mandatory before conducting any research involving animals or theses registration.

## Discussion

The present investigation aimed to document the challenges encountered in the establishment of the first IACUC in Egypt. At the start, a great number of the scientific community in the Cairo University, Faculty of Science were against its establishment citing the following arguments: 1) lack of guidelines that organize the work with animals in Egypt; 2) absence of animal welfare act regarding use of animals in research and teaching; 3) ill-equipped animal room to provide appropriate animal husbandry, care and use. Despite these issues, our determination enabled us to convince the decision makers, scientific colleagues, and other stakeholders of the inevitability of its establishment. Starting IACUC would help our resourceful scientists to publish their studies in acclaimed peer reviewed journals, to gain grants from international funds and institutions, and to form scientific cooperation with reputable universities worldwide. Furthermore, all these factors would help Cairo University in attaining a competitive rank amongst the world class institutions.

In support of the IACUC, we developed an application form and SOPs to regulate the reviewing procedures. To address the issues raised by our colleagues the following actions were taken: (1) With respect to lack of guidelines, the IACUC decided to follow the Guide for Care and Use of Animals (Guide, 2011); (2) the committee started to reorganize the animal house at the Department of Zoology to be compliant with the minimum requirements set by the international guidelines. The committee also developed a proposal to design and establish a state of the art animal facility in the Cairo University.

The few protocols submitted to the IACUC in 2013 may be attributed to lack of conviction and mandatory regulation for having an IACUC approved study, and low percentage of research paper rejection due to absence of approval by acclaimed peer reviewed journals. In 2014, the faculty board decided that the approval of the IACUC of the research study using animals is a condition for registration for M.Sc and Ph.D., degrees, in addition to the stipulation of an ethical approval for publication of the study.

The committee approved all protocols in 2013 to encourage and train the researchers as well as the IACUC members and to develop an adequate application form and a well-organized system. In 2014, the percentage of rejections and deferrals increased as the numbers of protocols increased and experience of the members also increased. Regarding the types of reviewed protocols, it was noted that the majority of the total reviewed protocols were those of MSC degree and the minority were research papers; this may be attributed to the obligatory approval by the ethics committee prior to registration.

The performance of the committee members has improved considerably after attending PRIM&R conferences. The IACUC was able to acquire basic administrative facilities such as an office, computer with an internet access, a copier and a well trained secretary. The committee also identified the importance of the presence of policies that regulate handling of the experimental animals and developed guidelines that help investigators when performing their research. There is no system for accreditation or assessment of IACUC in Egypt, but the members believe that accreditation of programs improve the quality of ethics review by encouraging the development of standardized policies and procedures, promoting a common base of knowledge, and enhancing the status of research ethics committees within their own institutions [14], thereby it will seek international accreditation.
